# Simcryocluster: a semantic similarity clustering method of cryo-EM images by adopting contrastive learning

**DOI:** 10.1186/s12859-023-05565-w

**Published:** 2024-02-20

**Authors:** Huanrong Tang, Yaowu Wang, Jianquan Ouyang, Jinlin Wang

**Affiliations:** Department of Computing, Xiangtan University, Xiangtan, China

**Keywords:** Cryo-EM, Protein structure determination, Contrastive learning, 2D classification

## Abstract

**Background:**

Cryo-electron microscopy (Cryo-EM) plays an increasingly important role in the determination of the three-dimensional (3D) structure of macromolecules. In order to achieve 3D reconstruction results close to atomic resolution, 2D single-particle image classification is not only conducive to single-particle selection, but also a key step that affects 3D reconstruction. The main task is to cluster and align 2D single-grain images into non-heterogeneous groups to obtain sharper single-grain images by averaging calculations. The main difficulties are that the cryo-EM single-particle image has a low signal-to-noise ratio (SNR), cannot manually label the data, and the projection direction is random and the distribution is unknown. Therefore, in the low SNR scenario, how to obtain the characteristic information of the effective particles, improve the clustering accuracy, and thus improve the reconstruction accuracy, is a key problem in the 2D image analysis of single particles of cryo-EM.

**Results:**

Aiming at the above problems, we propose a learnable deep clustering method and a fast alignment weighted averaging method based on frequency domain space to effectively improve the class averaging results and improve the reconstruction accuracy. In particular, it is very prominent in the feature extraction and dimensionality reduction module. Compared with the classification method based on Bayesian and great likelihood, a large amount of single particle data is required to estimate the relative angle orientation of macromolecular single particles in the 3D structure, and we propose that the clustering method shows good results.

**Conclusions:**

SimcryoCluster can use the contrastive learning method to perform well in the unlabeled high-noise cryo-EM single particle image classification task, making it an important tool for cryo-EM protein structure determination

## Background

In the life sciences, the structure of living organisms determines the function, and the three-dimensional structure of organisms is becoming more and more important for the basic research and application of life sciences. Structural biology methods mainly include X-ray crystallography (X-ray crystallography) [1], nuclear magnetic resonance spectroscopy (NMR) [2] and cryo-electron microscopy (Cryo-EM). In recent years, technological advances in sample preparation, computation, and especially instrumentation have made the single-particle cryo-EM method increasingly important in the field of structural biology. In Cryo-EM, in order to construct a high-resolution 3D reconstruction of protein structure using Cryo-EM technology, hundreds of thousands of single-particle images extracted must be accurately 2D classification [3]. 2D classification is an important intermediate stage in cryo-EM 3D reconstruction [4], and the class average results obtained at this stage can be used both as a template for single-particle selection and as the basis for the construction of subsequent 3D initial models [4, 5]. In the sample preparation process, in order to avoid high dose electron beams causing radiation damage to the sample and destroying atomic covalent bonds, low-dose electron beam imaging is generally selected, which results in a very low SNR of the obtained micrographs [6]. In addition, for single-particle low-electron beam imaging in the free state, it is impossible to manually label according to the projection angle, and the data with real labels cannot be obtained, so that the deep learning classification model that currently performs well in supervised classification tasks can not be directly applied in Cryo-EM single-particle image classification, and it is difficult to evaluate the quality of the classification results. Aiming at the above problems, it is of great significance to find a high-precision and effective two-dimensional classification method for the results of three-dimensional reconstruction.

Over the past few decades, many different approaches have been proposed for 2D classification of cryo-EM single-particle images. The main methods of unsupervised 2D classification that are currently popular, the following are: Cross-correlation (CC) and multivariate statistical analysis (MSA) enables K-means clustering with reference-free alignment [7, 8], Unsupervised maximum likelihood (ML) or maximum posterior (MAP) classification [9], statistical manifold learning algorithm (ROME) [10] for unsupervised single-particle deep clustering, variational self-encoder (VAE) [11–14] and multi-reference alignment (MRA) classification [15, 16]. The first two are traditional unsupervised classification methods, and the latter two are reference-free clustering methods based on deep learning. In the first method, the classification accuracy is affected by the noise-induced misalignment resulting from false peaks in cross-correlation calculation. Noise in single-grain images also affects the calculation of distances in k-means clusters. When the SNR is reduced, the performance of this classification method is also degraded. Compared to the K-means method, the ML-based method explores the optimal probability of measuring image similarity and exhibits good robustness in noisy single-particle image alignment tasks. A key problem is that the likelihood matching insufficiently differentiates structural heterogeneity among similar but critically different views. In each set of results after classification, due to structural heterogeneity, the number of valid categories is low, while increasing the cycle of ML optimization. In order to overcome the shortcomings of the above two traditional reference-free classification methods, Jiayi Wu et al. [17] proposed a statistical manifold learning algorithm for unsupervised single-particle deep clustering. The algorithm can effectively detect the structural differences between classes and classes, and improve the detection accuracy. However, there is still a lot of room for improvement in accuracy on high-noise images. Subsequently, Guowei Ji et al. [11] proposed a classification method based on variational autoencoders (VAE) and multi-reference alignment (MRA) to complete 2D classification. This method first uses VAE for noise reduction, and then uses the MRA-based K-means algorithm for unsupervised clustering, which can effectively process electron microscopy single-particle images under low SNR. Vignesh Prasad et al. [12] proposed an image clustering method using VAE and GMM priors, which jointly learns the prior and posterior and in turn learns a latent space representation for accurate clustering. This method does not require pre-training and is the first fully unsupervised VAE image clustering method. Nina Miolane et al. [13] combined VAE and GAN to learn the latent space of cryo-EM images, where the encoder encodes the image into a latent variable and the decoder decodes it into a reconstructed image, while the discriminator determines the probability that the input image is a real image. This method can compute the orientation and camera parameters of a given image. Alireza Nasiri et al. [14] proposed a translational and rotational group-equivariant variational autoencoder architecture, which enables learning of translation and rotation-invariant object representation in images in an unsupervised manner. However, the performance of this method on the real particle image of cryo-EM still needs to be strengthened.

Based on the shortcomings of the above methods, we propose a low SNR single-particle image classification method based on contrast learning, which performs well in both the simulation dataset and the real data set. The main contributions of this article are as follows: (1) In this paper, a cryo-EM clustering model based on contrast learning is proposed, which is used to complete the feature extraction task of unlabeled cryo-EM images, calculate similar features to generate pseudo-label data, and then complete the classification. (2) This paper proposes to use the frequency domain spatial interpolation method for efficient alignment in each set of data after the classification is completed, and then complete the class average calculation.

In this study, we divide the task into three steps. The first is preprocessing, then using the deep learning model to develop a good feature extractor for feature extraction, and finally to make learnable clustering of the extracted features. The method in this paper trained and tested three datasets of 80 S ribosome, superpolarized cyclic nucleotide HCN1, polypeptide toxin and gummolycer glyceride toxin complex TRPV1, and in the 80 s ribosome verification experiment, the ACC could still reach 78.59 at the SNR=0.1, thus demonstrating the effectiveness of this method.

## Methods

**Fig. 1:**
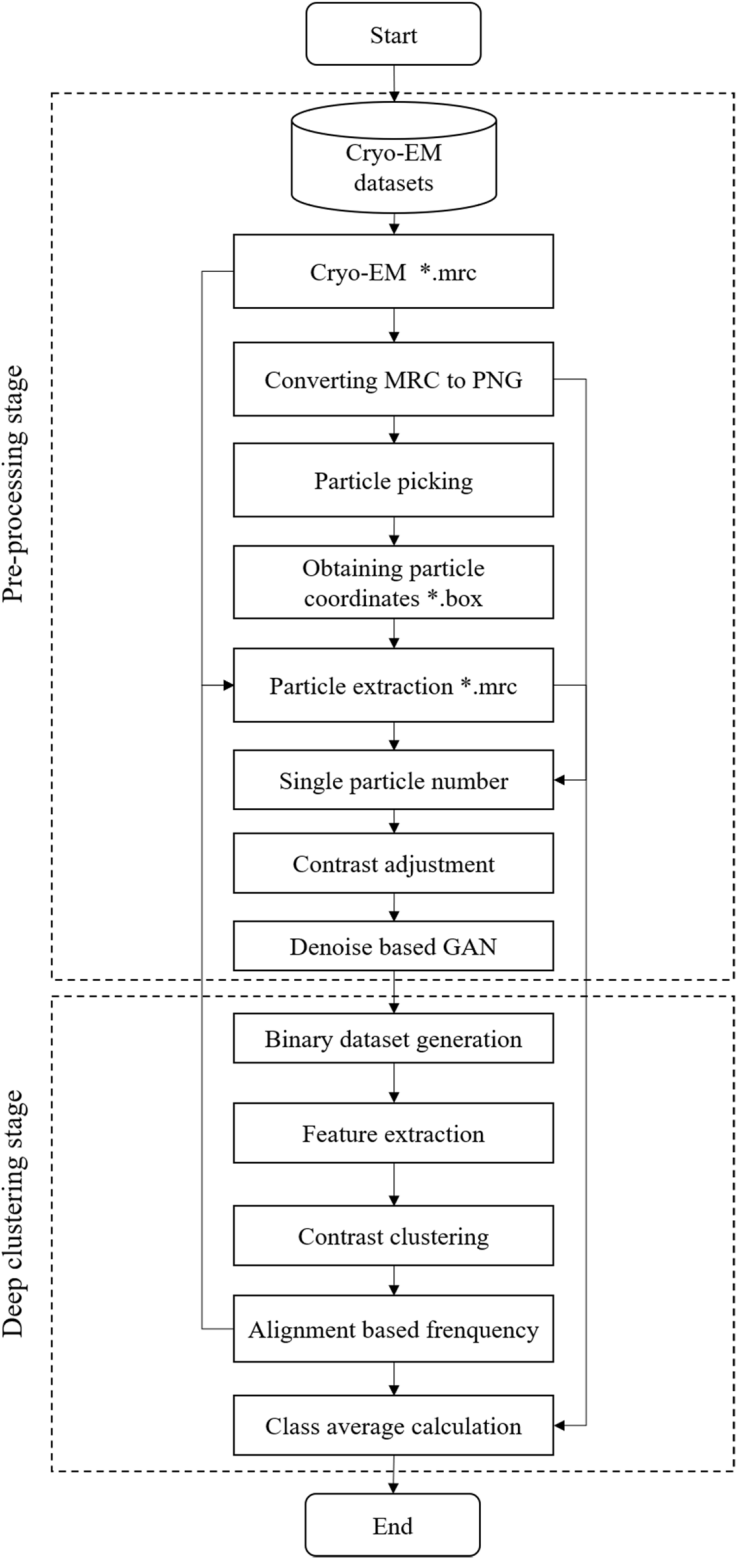
SimCryoCluster workflow. The general framework of SimCryoCluster: Learning single-particle clustering. The dotted box represents the two phases of the method: preprocessing and particle depth clustering. A solid box denotes an analysis step

Our SimCryoCluster framework for 2D classification of cryo-EM single-particle images is shown in Fig. 1. In this framework, there are two main parts of work, the first part is the data preprocessing part, and the second part is the image clustering part, in which we propose a new method, which mainly uses a contrast learning model to complete the unsupervised clustering task of feature extraction of single-particle images, which can be completed without considering the data distribution. This process consists of four main stages: data preprocessing, feature extraction based on contrast learning, learnable clustering and reference alignment within the frequency domain space class. In preprocessing stage, several image processing methods are applied to enhance the input cryo-EM single particle image such as denoising base on GAN, Contrast Enhancement Correction (CEC),etc. Feature extraction is the use of contrast learning networks to extract feature vectors to reduce the dimensionality of data, similar to sPCA. Without labels classification is to maximize the similarity of feature vectors extracted by the network, calculate k near neighbors of each sample, and store the neighbor characteristics of each sample for subsequent learning clustering tasks. Alignment within the frequency domain spatial class is an invariant feature of estimating the rotation of an image, performing in-plane rotation and translation alignment on classified data in Fourier space.

### Feature extraction and contrast clustering

**Fig. 2:**
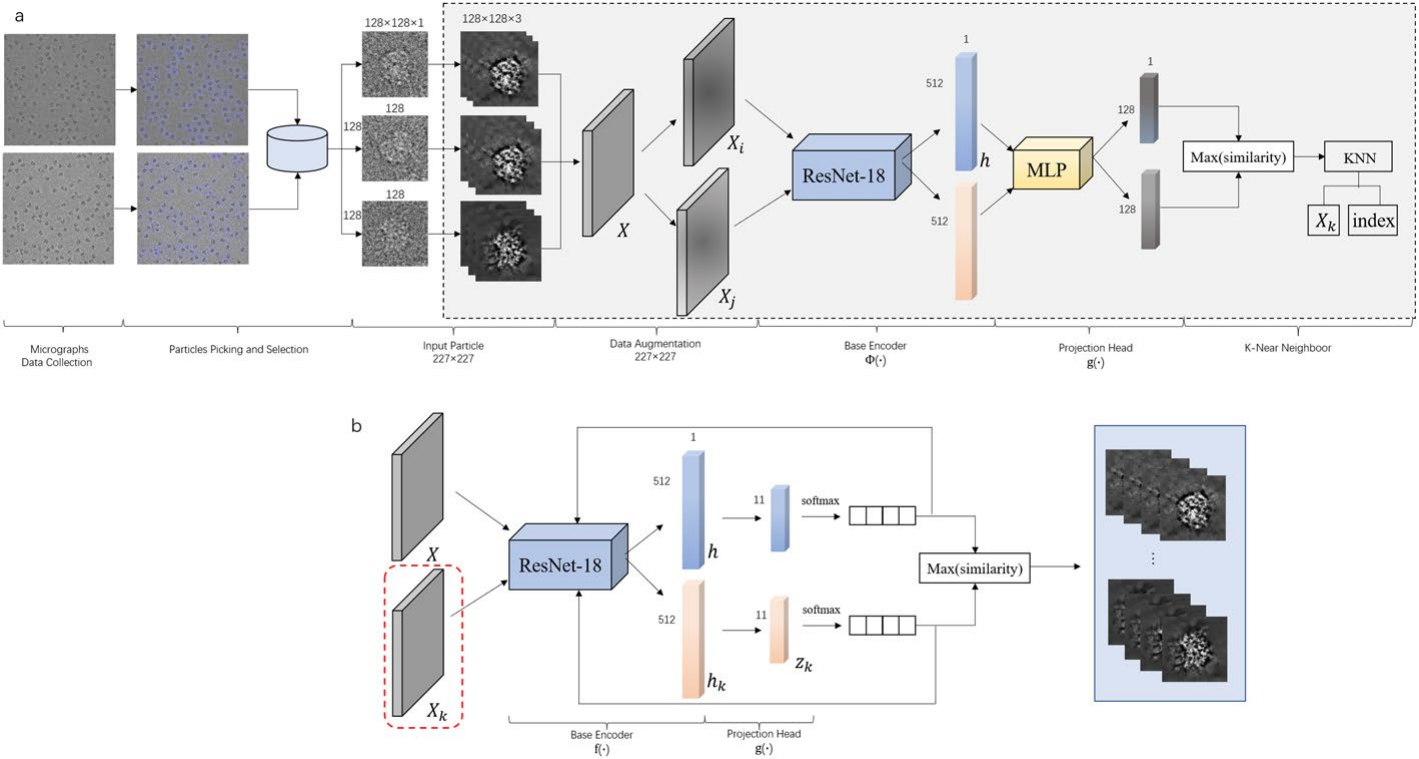
The overall framework of a clustering network. **a** Cryo-EM image features are extracted to generate K near neighbors. **b** Fuse the output of a for clustering

Due to the random angle, noisy noise, and unknown distribution of cryo-EM single-particle images, methods based on maximum likelihood do not yield good results. In such a low SNR scenario, how to obtain effective features and improve clustering accuracy is a key issue in the analysis of single-particle data of cryo-EM. A label-free classification network based on contrast learning is constructed on the basis of this problem, which in turn accomplishes 2D classification. Before the electron microscopic single particle images are fed into the feature extraction network, data enhancement of the input data will be performed as a pre-task. First, for each image in the dataset, two enhancement combinations are performed (i.e., crop and resize and recolor, resize and recolor, crop and recolor, and many other combinations). The two enhanced images are essentially different versions of the same image. The two images are fed into the feature extraction network model, and each image generates a corresponding feature vector, with the goal of training the model to output a similar representation of a similar image. Details can be showed on Fig. 2.

For each original image X, through data enhancement, transform into $X_i$ and $X_j$, and then by training the base encoder network $f(\cdot )$ to obtain the feature vector of the enhanced image, and then use a small neural network projection head $g(\cdot )$ Map representations to the contrast loss space and maximize the contrast loss to ensure consistency of features after network training. After the training is complete, we discard the projection head $g(\cdot )$ and use the encoders $f(\cdot )$ and the representation h for the downstream task. where ${\varvec{h}_{i}=f\left( \tilde{\varvec{x}}_{i}\right) =ResNet({\tilde{\varvec{x}}_{i})}}$, where ${\varvec{h}_{k} \in R^{d}}$ represents the output after averaging pooling. $h_i$ is the sample feature output after using the backbone network *f*, and $z_k$ is the feature after the projected output terminal *g*.

Since this task needs to output the image as 11 classes, the 512-dim feature is reduced to an 11-dim feature vector through a linear layer, and then the eigenvector is converted to a probability vector by the softmax layer, and then the cosine similarity function is used to calculate the similarity of the probability vector to obtain the final clustering result. The main purpose of using probability vectors is to generate pseudo-labels by calculating the probability values in the classification, and when the probability is greater than 0.95, it is set as a pseudo-label, and feedback is given to train the network again to update the weights.

### Alignment based on frequency domain space

**Fig. 3:**
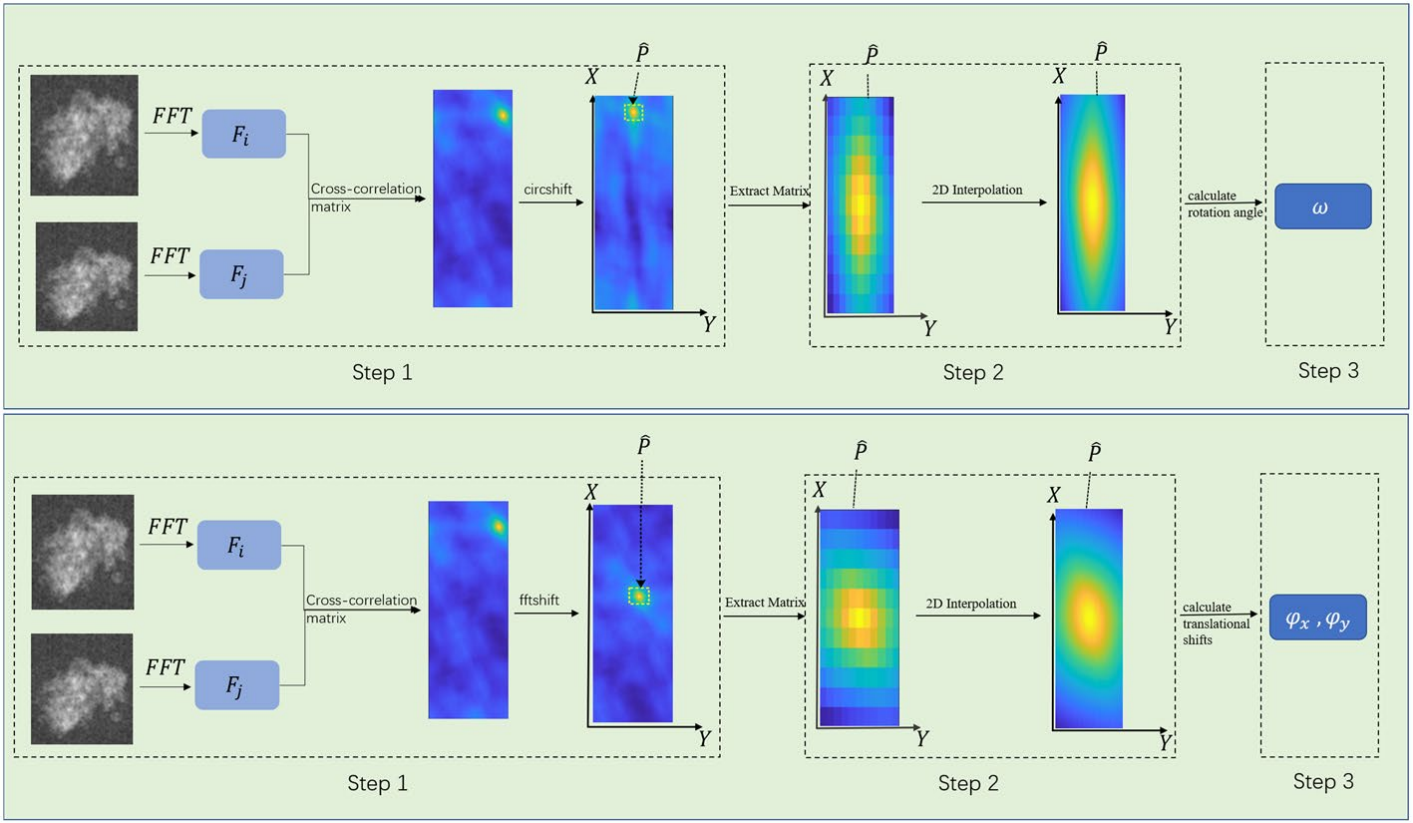
Schematic diagram of rotational alignment and translation method for maximum near-neighbor interpolation in frequency domain space. **a** Image rotation alignment. **b** Image translational alignment

Image alignment is a basic and essential step in the 2D classification task of cryo-EM single-particle images [18, 19]. The purpose of image alignment is to estimate the three parameters of alignment, namely the angle of rotation, and the translational movement in the direction of the x- and y-axes. Image rotation alignment and translation are also often used in time domain space, but in time domain space it is usually matched by rotation in a certain step, it takes multiple iterations to calculate the alignment parameters, and the result is an integer [20]. In frequency domain space, the calculation alignment parameters can be calculated directly without enumeration. On this basis,we use an alignment algorithm based on two-dimensional neighbor interpolation in the frequency domain of the image, which can improve the accuracy of the estimated parameters. The specific steps can be divided into rotational alignment and translation alignment, for the calculation of rotational alignment, first of all, the two images in the class are parallel fast Fourier transform (PFFT), the cross-correlation matrix of the two images is calculated, positioned to the maximum value in the matrix, two-dimensional interpolation around the maximum value, and the rotation angle between the two images can be directly determined according to the position of the maximum value in the matrix. For the calculation of translation alignment, only two images need to perform a fast Fourier transform (FFT) on it. In the single particle selection, usually use a certain radius size of the circle for selection, when extracting (Extract), the extraction box is usually selected not less than the diameter of the circle square box for frame. Therefore, the size of the single-grain image we are dealing with can be set to $n\times n$, and the rotational alignment used in this article is based on the square image. The main process of rotation alignment is to calculate the cross-correlation matrix, complete the two-dimensional interpolation of the nearest neighbor, and finally calculate the rotation angle, a total of three steps, the specific process can be seen in Fig. 3.

First of all, suppose that the input two images are $N_i$, $N_j$, through the parallel fast Fourier transform can obtain the two images related spectrogram $F_i$, $F_j$, its size is $(n/2)\times 360$, by calculating the spectrum map to calculate the cross-correlation matrix P, the specific calculation such as Eq. 1.

1 $$\begin{aligned} P={\text {abs}}\left( i f f t \left( F_{i} \times {\text {con}} \,j\left( F_{j}\right) \right) \right) \end{aligned}$$

where $conj(\cdot )$ denotes the computation of the complex conjugate function. $ifft(\cdot )$ denotes the two-dimensional fast Fourier inverse transform $abs(\cdot )$ denotes the absolute value function, and all three functions can be represented in MATLAB. The values in the reciprocal matrix P need to be cyclically shifted by *m*/4 positions to exchange the horizontally centered maximum value, and the function for shifting the values in the matrix can be implemented using the $'cirshit'$ function in MATLAB.

A two-dimensional interpolation occurs near the maximum value of the cross-correlation matrix. the angle of rotation of the image $N_j$ relative to the $N_i$ that can be determined based on the position of the maximum value in the cross-correlation matrix P on the x-axis. The $\omega$ value calculated here is an integer. First, first find the maximum value of the cross-correlation matrix P, interpolate based on the nearest neighbor of the maximum value, that is, extract the maximum value from the matrix of the central matrix $\widehat{P}$, as shown in the red line in Fig. 9, two-dimensional interpolation in the $\widehat{P}$ matrix, the specific function can refer to the $'interp2'$ function in MATLABL.

The final is to calculate the rotation angle, according to the position of the maximum value in the matrix P after x-axis interpolation, the rotation angle $\omega$ can be directly calculated, where $\omega \in [-\pi , \pi ]$. For a better representation of the angle of rotation, adjust it to a positive integer as specifically shown in Eq. 2.

2 $$\begin{aligned} \omega =\left\{ \begin{array}{ll} \omega , &{} \text{ if } 0 \le \omega \le \pi \\ \omega -2 \pi , &{} \text{ if } \pi<\omega <2 \pi \end{array}\right. \end{aligned}$$

### Class averaging

In order to further improve the SNR of cryo-EM single-particle images, the results of each class are averaged after classification, and the class average plot is obtained. To improve the result of the class average, we discarded the traditional direct averaging method and chose the weighted average, which is based on the probability vector of the softmax output in Fig. 8b, $[p_1, p_2,... p_i]$ determines the outcome of each class, and saves the probability values of the corresponding category, normalizes all the probability values of the final category to obtain the corresponding weight w, multiplies each image in $I_j$ the same category by the weight w,among $0 \le \omega < 1$. And then adds up to obtain the final class average result. The final result is shown in Fig. 6b.

## Results

### Construct simulation dataset

Since the original cryo-EM single-particle images without label data cannot be labeled by experts, this poses a huge challenge to the evaluation of 2D classification results in 3D reconstruction. Aiming at the above problems, We constructed a simulated cryo-EM dataset by using Scipion to perform 2D projection of particles and adding real noise to the resulting images. [21]. When obtaining the projection map, we first selected three protein macromolecules with PDB structures from the electron microscopy database (EMDB) [22], corresponding to IDs of 3j7a, 5u6o and 5irx, and their specific parameters are shown in Table 1. Then, Use the XMIPP [23] software processing package to simulate the effects of a real microscope. The projection is mainly based on the rotation angle (rot) and tilt angle transformation, the step size of both is set to 5$^{\circ }$ when the data is constructed, and the rotation angle is projected in Eq. 3 when rotating, and the change in the tilt angle is the same as above.

3 $$\begin{aligned} \theta = \frac{rot_{F}-rot_{0}}{rot_{Step}} \end{aligned}$$

where ${rot}_0$ represents the minimum, maximum ${rot}_F$ and step value ${rot}_{Step}$ of the rotation angle, the rotation angle range is from 0$^{\circ }$ to 360$^{\circ }$ in degrees, tilt is also in degrees, the range is 0$^{\circ }$ to 180$^{\circ }$, when $tilt=0$ represents the top view, $tilt=90$ represents the side view. We generated 1100 projected images for each protein, covering 11 different horizontal rotation angles (grouped within 5$^{\circ }$), i.e. 100 projected images per rotation angle.

**Table 1:** Sample dataset detailed parameters

Criteria	80S ribosome	TRPV1 complex with DkTx and RTX	HCN1
Data ID	EMPIAR-10028	EMPIAR-10059	EMPIAR-10081
PDB ID	3j7a	5irx	5u6o
Image size	181$\times$181	104$\times$104	104$\times$104
Voltage (KV)	300	300	300
Average electron dose	20	41	1.26
Nominal CS (mm)	2.0	2.0	–
Defocus ($\mu$m)	0.8$\sim$3.8	0.7$\sim$2.2	1.5$\sim$3.3
Pixel size (Å)	1.34	1.22	1.30
Particle size (pix)	360	192	256

### Fusion analog noise

**Fig. 4:**
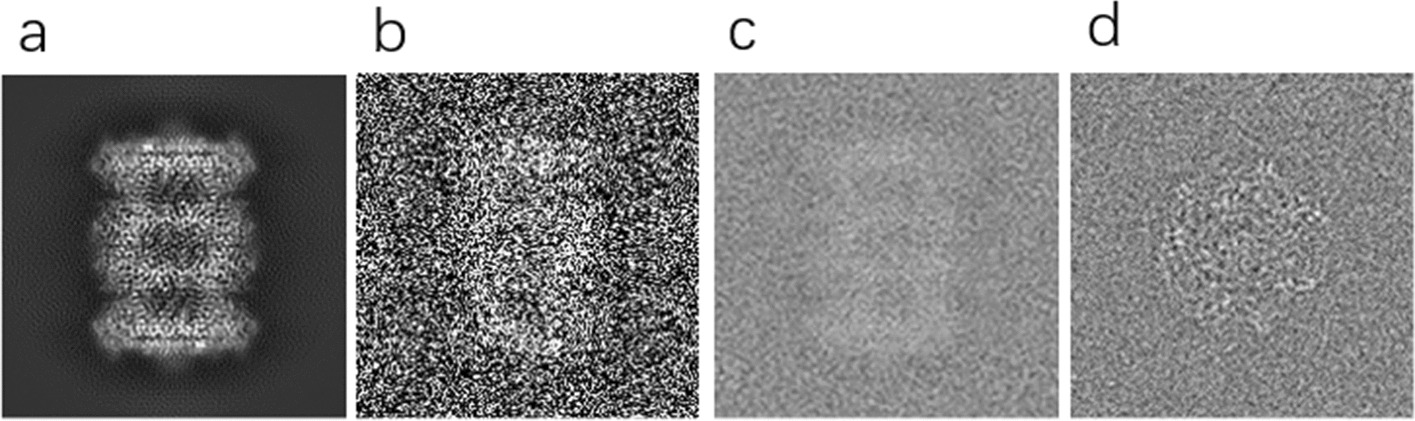
Building a visual comparison of the simulated dataset. **a** Realistic projection image. **b** Visualize the results after noise reinforcement by Gaussian white. **c** Visualization of the result by overlaying the extracted noise blocks by a projection plot. **d** Realistic label-free particle cryo-EM images

In previous methods of constructing simulation datasets, researchers typically used Gaussian white noise to simulate cryo-EM noise. However, real cryo-EM data noise is difficult to obtain in reality or the noise distribution is difficult to derive. In general, we cannot obtain cryo-EM images with known noise distribution. In real life, the noise of these unknown noise images is very complex and the distribution is unknown, so using existing models trained on a particular noise does not yield good results [24]. In this paper, the U-Net network is used to split the noise block and the pixel block, and the extracted noise block is superimposed with the projection map, and the simulated noise single particle image corresponding to the clean particle is constructed, which can be used for subsequent network model training [21]. The visual comparison of the dataset construction is shown in Fig. 4.

### Performance evaluation metrics

To better evaluate the results of this method on the simulated dataset, we used accuracy (ACC) to evaluate the results of the feature extraction phase and Fowlkes-Mallows index (FMI) to evaluate clustering performance [25],such as Eq. 4.

4 $$\begin{aligned} A C C(\omega , C)=\frac{1}{N} \sum _{k} \max _{j}\left| I_{k} \cap c_{j}\right| \end{aligned}$$

where $\omega$ represents the set of results after the k-nearest neighbor classification is calculated by feature extraction, $\omega =\left\{ I_1,I_2,\ldots ,I_j\right\}$, A collection of real label datasets $\textbf{C}=\left\{ c_1,c_2,\ldots {,c}_k\right\}$. The results of the classification after the completion of feature extraction may not match the original label data at the time of verification, which will lead to lower ACC results,such as Eq. 5. When validating the results, this paper uses the Kuhn-Munkres algorithm to calculate the maximum match.

5 $$\begin{aligned} FMI = \frac{T P}{\sqrt{(T P+F P)(T P+F N)}} \end{aligned}$$

where *TP* is the number of particles correctly classified in the total image of a single particle, *FP* is the number of particles that are misclassified, and *FN* is the number of particles that are incorrectly predicted as incorrect. By simulating the dataset, the real live labels can be effectively recorded according to the images of different projection angles.

### Data pre-processing

#### Step 1: Voxel image conversion

The acquired raw cryo-EM images are stored in Mixed Raster Content (MRC) format, which defines a three-dimensional grid (array) of voxels, each with a value corresponding to the electron density or potential. In order to facilitate preprocessing and improve the SNR, we converted the cryo-EM single particle image mrc format to the commonly used 16-bit PNG format. We preprocess the simulated dataset and the real dataset separately. In order to facilitate the processing of real datasets, we name all files in ascending order during format conversion, so that the results after classification can be output according to the file name index. Our goal is to extract the feature information of the single particle image through the contrastive learning network, compare the features with similarity, and obtain the clustering results. Therefore, in order to make the network model learn better, we choose the denoising method based on the generative adversarial network (GAN)to improve the quality of cryo-EM single particle images. In addition, we perform contrast adjustment on the transformed image.

#### Step 2: Contrast adjustment

Since the low-dose optical imaging module during cryo-EM imaging is on the defocused particle region, the obtained single-particle image has a low-contrast property that is difficult to identify. Histogram equalization based on uniform distribution can be used to increase the intensity value of image pixels [26]. It increases and improves global image contrast by mapping the original image histogram to a unified histogram. Therefore, in order for subsequent network models to learn better, we perform contrast adjustments on the images.

#### Step 3: Single-particle images denoising

**Fig. 5:**
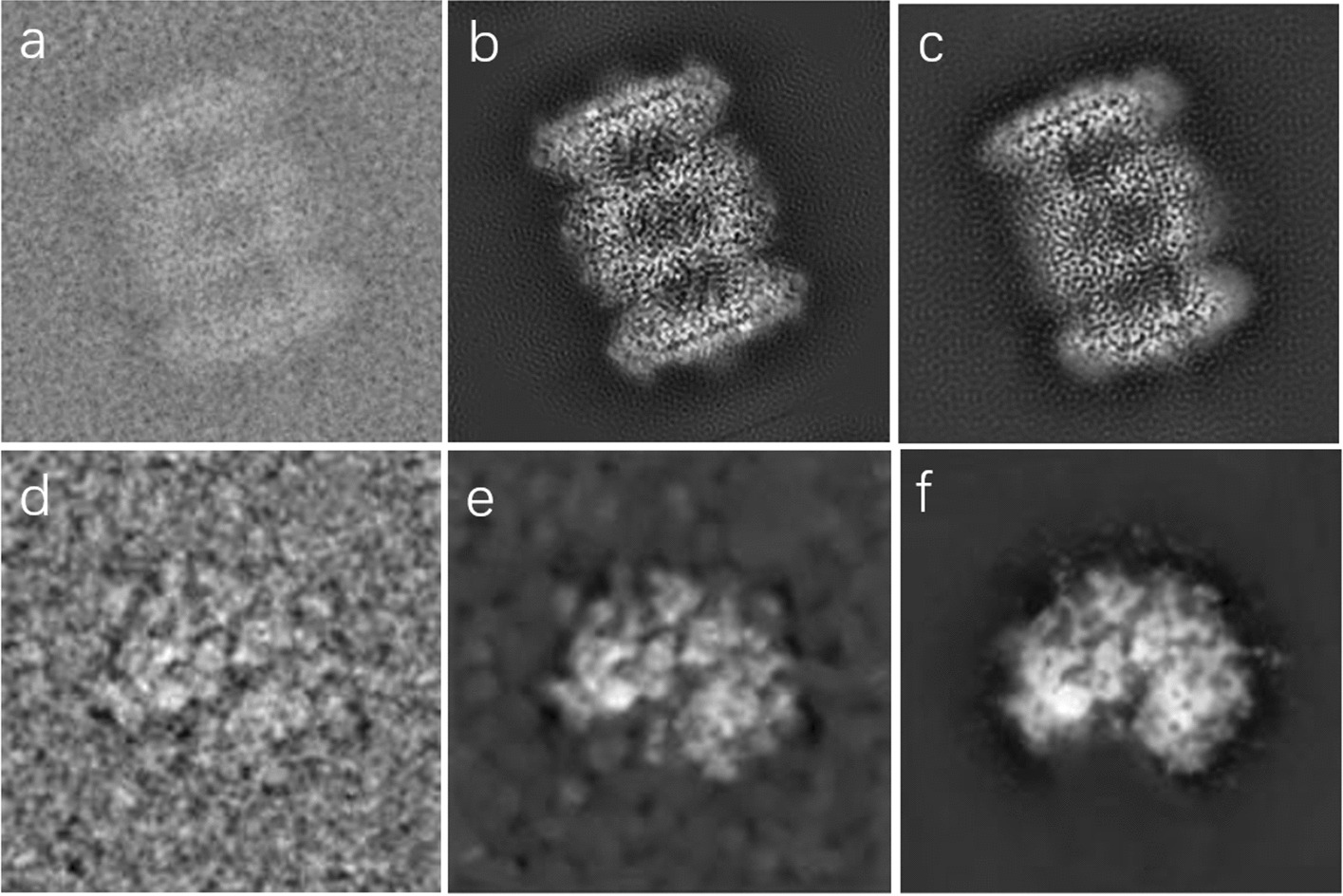
Modified Generative Adversarial Network (GAN). **a** Original cryo-EM single-particle simulation image from the T20s proteasome. **b** A true noise-free image with a central section of the EMD-3347 PDB structure can be used as a label sample for denoising image a. **c** The result of label noise reduction by means of an improved GNA method. **d** Original cryo-EM single-particle image from the Plasmodium falciparum 80 S ribosome (EMPIAR-10028). **e** Represents the result of denoising the Original cryo-EM single-particle image using a GAN. **f** It shows the denoising results using the improved GAN method of Tang et al. [21]

Due to cryo-EM imaging, electron beam electron doses are small, and the contrast between proteins and solvents is low and noisy. Image recovery techniques are commonly used for cryo-EM single-particle image denoising. Based on prior knowledge of the noise reduction process, image recovery recovers and improves image quality by identifying the type of noise and then eliminating it. Therefore, we chose Tang et al [21], the proposed improved denoising algorithm for generating adversarial networks (GANs) [27], In the architecture of the GAN network, the generator network adopts a symmetric structure that consists of three blocks: the convolution block, the residual block, and the sub-pixel convolution block. The discriminator employs a convolution network with five layers, including batch normalization layers and LRelu layers. The training dataset for this network uses a simulated dataset constructed with various particle images, and the network is tested with the EMD-0406 dataset and EMD-23579 dataset, each with added noise blocks of varying levels. which preserves as much protein internal conformational information as possible while reducing noise,,We use relion to particles picking from Plasmodium falciparum 80 S ribosome and EMD-3347, and conduct denoising experiments on the particle picking results. which improves the quality of cryo-EM single-particle images as shown in Fig. 5.

### Construct network training and test

In order to evaluate the effect of the network model used in this paper in the process of feature extraction and clustering, we construct a simulation dataset to verify the proposed method. We assume that the particles in all single-grain images in the simulation dataset are active particles, select 11 directions with different projection angles, divide all particles into 11 classes, and randomly rotate each class to obtain a 2900 single-grain image dataset. We split all single-particle datasets into training sets and validation sets, of which about $70\%$ were used for training sets (2900 particle images, 1800 for training and 200 for validation) and about $30\%$ testing (900 particle images). In addition,in order to avoid overfitting, the input image was horizontally flipped, randomly cropped, rotated, filled, etc. to expand the data set. The batch size was set to 128, the number of iterations was set to 500, and the model optimizer used SGD(Stochastic Gradient Descent), the learning rate adopts a dynamic adjustment strategy, and the initial learning rate is 0.4, We added different levels of noise to the training set and validation set, and the images with different signal-to-noise ratios of SNR=0.1 and SNR=0.6 are constructed as new data sets.

### Experiments on testing contrast learning classification models

#### Step 1: Experiments on feature extraction

**Fig. 6:**
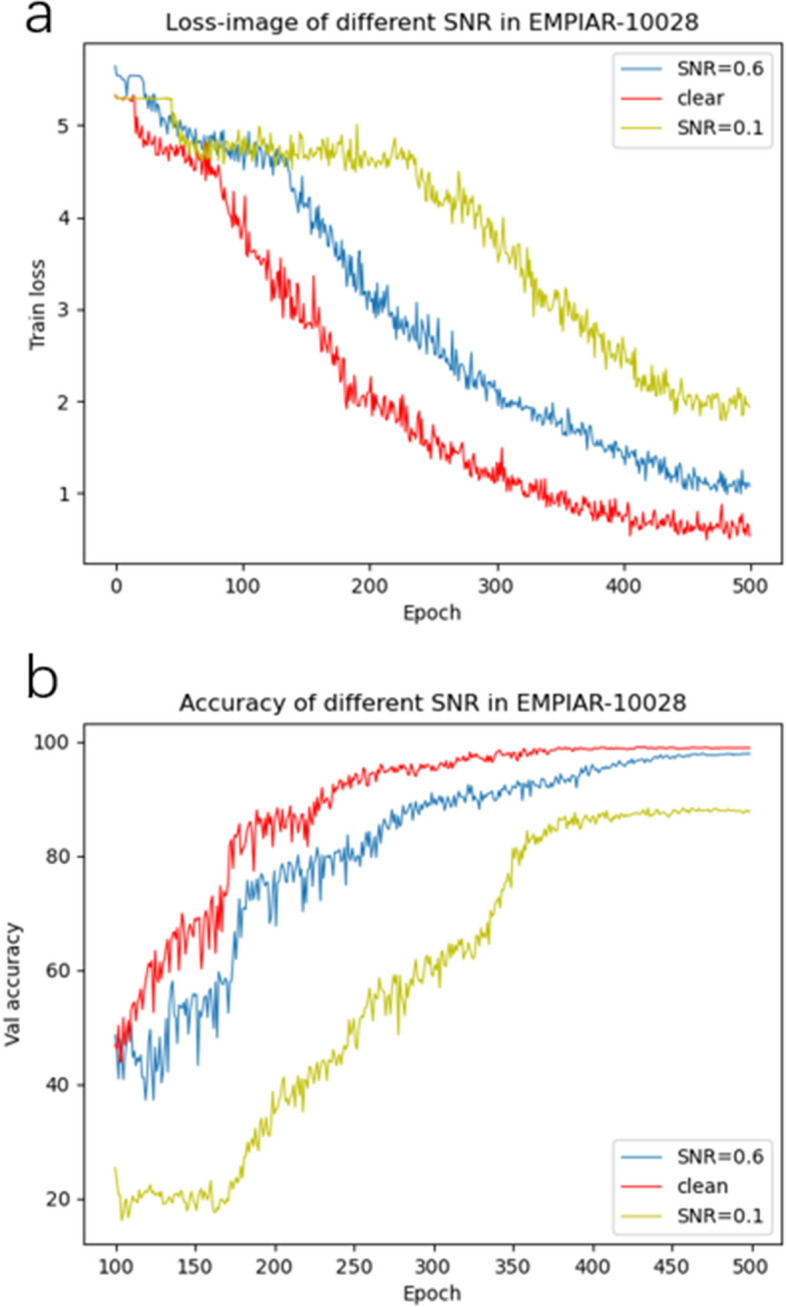
Loss function curves and KNN verification accuracy curves under different SNR conditions. **a** Loss function curves in the EMPIAR-10028 dataset under different SNR conditions. **b** KNN verification accuracy curves in the EMPIAR-10028 dataset under different SNR conditions

**Fig. 7:**
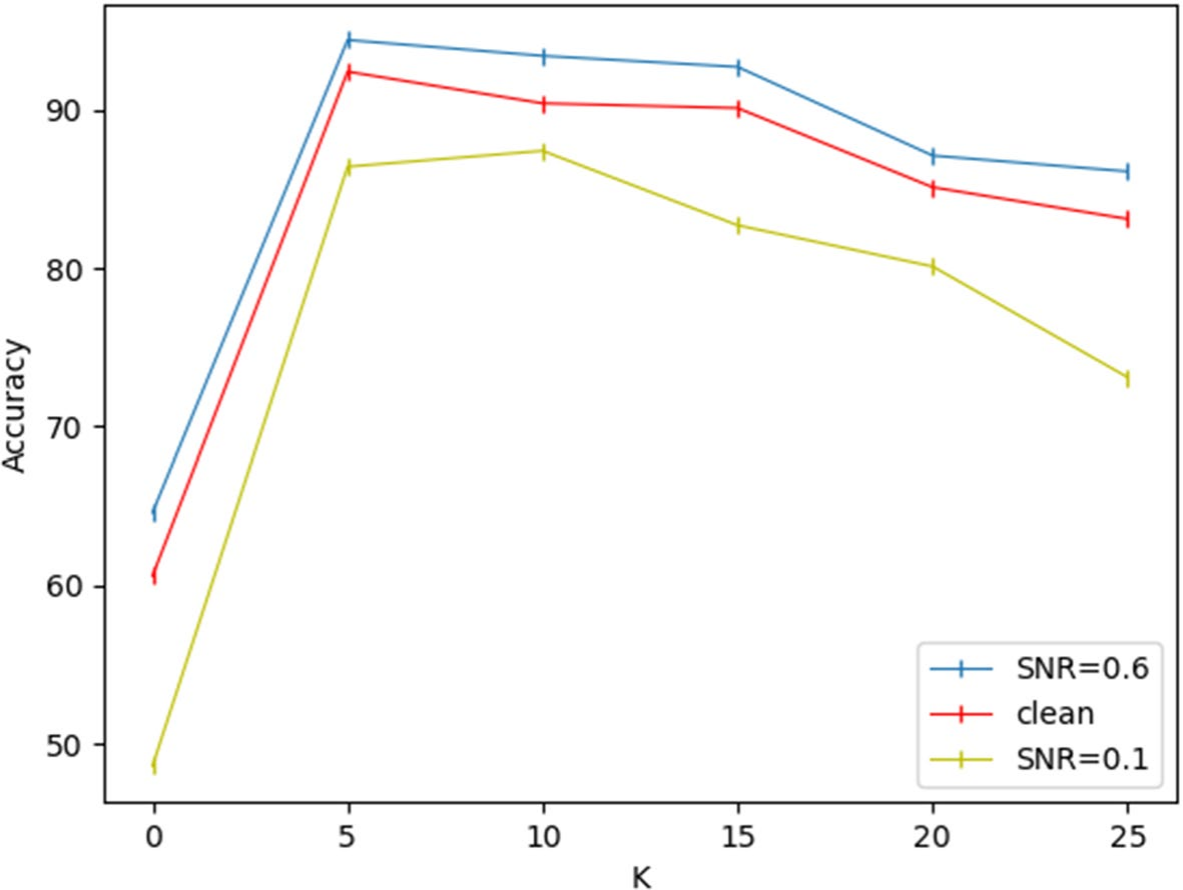
Influence of the used number of neighbors K

In the first stage, we will build the labeled training set and the validation set into a binary file, store the training set randomly in 5 $train-batch$, and store the test set in the $test-batch$. Labels are ignored during training based on contrast learning networks, and feedback training is performed on the network using augmented data from each data. Images of any size input can be converted to 128 $\times$ 128, which can be applied to network extraction features. Among them, Resnet-18 [28] is used as a backbone, and after network training, each image is converted into a 512-dimensional feature vector, and then the output 512-dim feature vector is fed into a multilayer perceptron (MLP) [29] and output as a normalized 128-dimensional feature vector. In order to verify the effectiveness of network feature extraction, the K near neighbor (KNN) method is introduced to calculate the neighbors of the normalized eigenvectors, and the corresponding labels are found according to the index, which can effectively calculate the verification accuracy after KNN classification, and store the K neighbors of each eigenvector in a temporary library, which can be used for the input of the second stage. In addition, the change of loss curve during training with the KNN verification accuracy curve can be seen in Fig. 6a, b. From the display of Fig. 6a, it can be seen that when the SNR is relatively high, the loss function as a whole is in a state of decline, and the characteristic information can be effectively learned. When SNR = 0.1, network training converges slowly, and after the first 200 epochs learn slowly, after 200 epochs, the Loss function begins to converge gradually until it is near 500 epochs, and the curve tends to flatten. From Fig. 6b, it can be seen that the accuracy of the final verification will be different under different SNR. In this process, with the network training, the learning and characterization ability of the network can be effectively improved, and the effective feature information can be obtained, so as to achieve better verification accuracy. When SNR=0.6, its top-5 accuracy tends to be about $93.1\%$ for noise-free particle images. When SNR=0.1, its top-5 accuracy is up to $87.92\%$. In this process, we have also gone through several experiments on the choice of K value, and it is found that the verification accuracy can reach the highest when K=5, as shown in Fig. 7. According to this validation accuracy, on the one hand, the validity of the first stage in feature extraction can be effectively proved. On the other hand, the meaningful nearest neighbors in the first stage can be integrated into the second stage of the learnable clustering method as a priori knowledge.

#### Step 2: Experiments on contrastive clustering

Through the first stage of training weights as the a priori input of the second stage, and then the network is retrained to obtain the feature input comparison loss function, the network model is continuously optimized, and a learnable clustering network is formed to complete the clustering task. BackBone uses the standard ResNet-18 [28]. For each sample, 10 nearest neighbors were identified by the instance discrimination task based on noise contrast estimation (NCE), and the clustering performance of the network could be effectively improved by fusing the near neighbor features in the first stage. In this paper, the EMPIAR-10028 dataset is experimentally verified under different SNR, and the clustering results are shown in Table 2.

**Table 2:** Validation set results for 11 classes at SNR = 0.6 in EMPIAR-10028

Metric	Top-1	Top-5	NMI
Pretext+K-means	0.57	–	0.63
SCAN	0.56	0.86	0.61
SimCryoCluster (Ours)	0.76	0.93	0.67

The results with K-means were obtained using the pretext features from Simclr [30]. We provide the results obtained by our baseline(SCAN) [31],and the results we have improved on the model

We compare our method with CL2D [32] and EMAN2 [33] on the simulation data of SNR = 0.1 and SNR = 0.6. In addition to these traditional methods, we also compared them with the classic convolutional autoencoder (CAE) and the improved iterative encoding method (IterVM), and the results are shown in Table 3.

**Table 3:** This article compares the 2D classification results with other methods on FMI scores with different SNR

Methods	SNR = 0.1	SNR = 0.6
CL2D	0.53	0.61
EMAN2	0.49	0.58
CAE	0.58	0.72
IterVM	0.65	0.82
SimCryoCluster (Ours)	0.71	0.85

### Experiments on alignment and class averaging

**Fig. 8:**
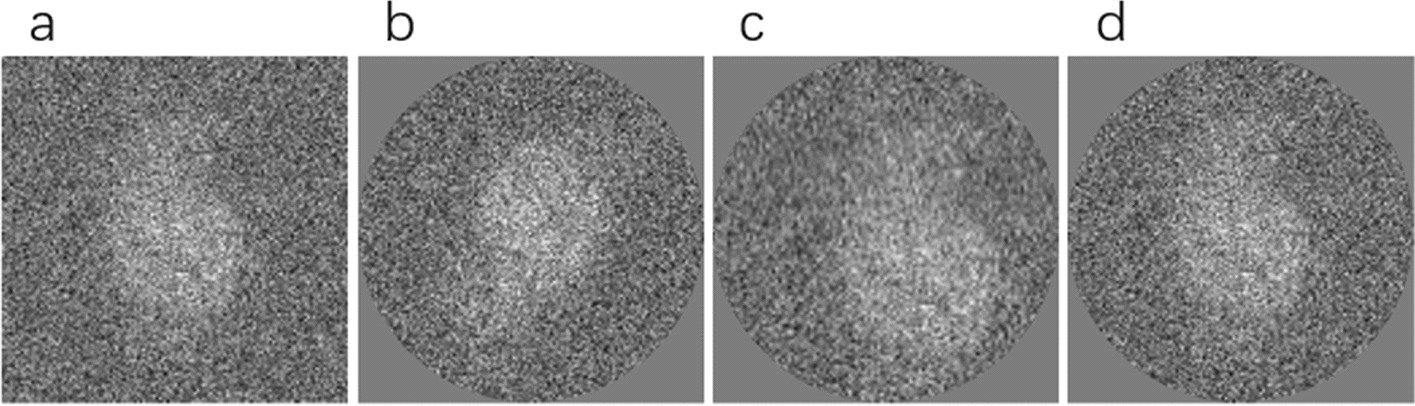
In-class rotation and translation alignment visualization results. **a** A single grain image is randomly selected from a certain category as a referenced original image. **b** Rotate only single-particle test images. **c** Shift single-particle test image. **d** Aligned single-particle image

The result of the classification can be calculated by the pixel mean, and a high-precision class-average image can be obtained, which is also one of the methods to improve the SNR of the image. However, the image needs to be aligned before the average pixel is calculated, and this paper proposes to use a fast frequency domain space-based maximum near-neighbor interpolation method that can estimate the rotation angle and translation alignment parameters in the x-axis and y-axis directions. The specific method implementation steps are in Alignment Based on Frequency Domain Space of the Methods. We randomly selected a class of results from the results after clustering EMPIAR-10028, randomly selected a particle from the class as a reference for reference alignment, and then manually adjusted to remove the wrong particles according to the index value, the process introduced manual intervention, which can effectively improve the calculation result of the class average. We used the traditional cryo-EM single particle reconstruction software (Relion) to conduct experiments because Relion has the characteristics of simple operation, clear process, and high reconstruction accuracy. Figure 8a–d is our class average result using relion, it show the results of the rotation and translation alignment visualization. The simulation experiments for both alignment and class averaging in this subsection were run on a six-core system with 24 GB RAM in a Windows 10 environment, on MATLAB R2019b.

### Performance comparison

Our SimCryoCluster classification model is compared to two mainstream 2D classification software (EMAN2 [33] and Relion3 [34]). Since multiple signal sources in the cryo-EM imaging process will make the cryo-EM image contain noise, and the SNR of the single-grain image obtained is low, the performance comparison is carried out in the case of SNR=0.1 and SNR=0.6, respectively, and the 80 s ribosome is selected as the data set, and the comparison results are shown in Table 4. Measuring the data running time and accuracy for different SNRs, SimCryoCluster achieved better classification results in the experiment, taking the least time to classify single particle projection images and achieving an accuracy of up to 94.20, indicating the effectiveness of our method.

**Table 4:** Performance test results of the simulated dataset on different classification methods

	Relion	EMAN2	SimCryoCluster
SNR = 0.6	0.83	0.78	0.94
SNR = 0.1	0.62	0.38	0.74
Times (h)	10	6	4.5

### Experiments on real datasets

**Fig. 9:**
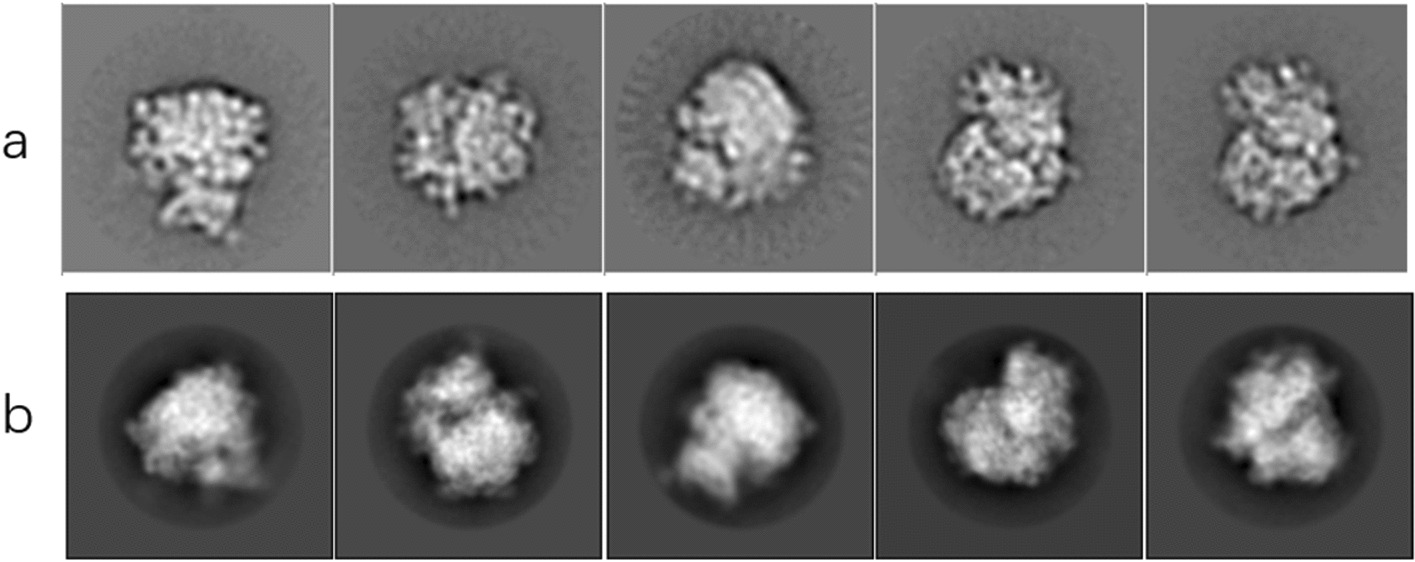
The final class average visualization result graph. **a** Visualize class averaging results by CL2D. **b** The average result of the visualization class after noise reduction by the method in this paper

In order to further verify the effectiveness of the deep clustering method proposed in this paper on the cryo-EM single-particle image, we selected 5000 single-particle images automatically selected from the original cryo-EM image, and entered the network for clustering and alignment after noise reduction. We use cl2d of Scipion software to compare with the relion classification experiment that incorporates our denoising results. We divide it into 20 classes, weight the average of the single-particle images in each class according to SNR, sort the class average results, and select the visualization results of the first five valid classes as shown in Fig. 9b. where Fig. 9a is a visualization of class averaging using CL2D. From the visualization results, it can be seen that the results of the class averaging after noise reduction can effectively retain important particle information, and the noise is significantly lower than that of CL2D clustering.

### Reconstruct the experiment

**Fig. 10:**
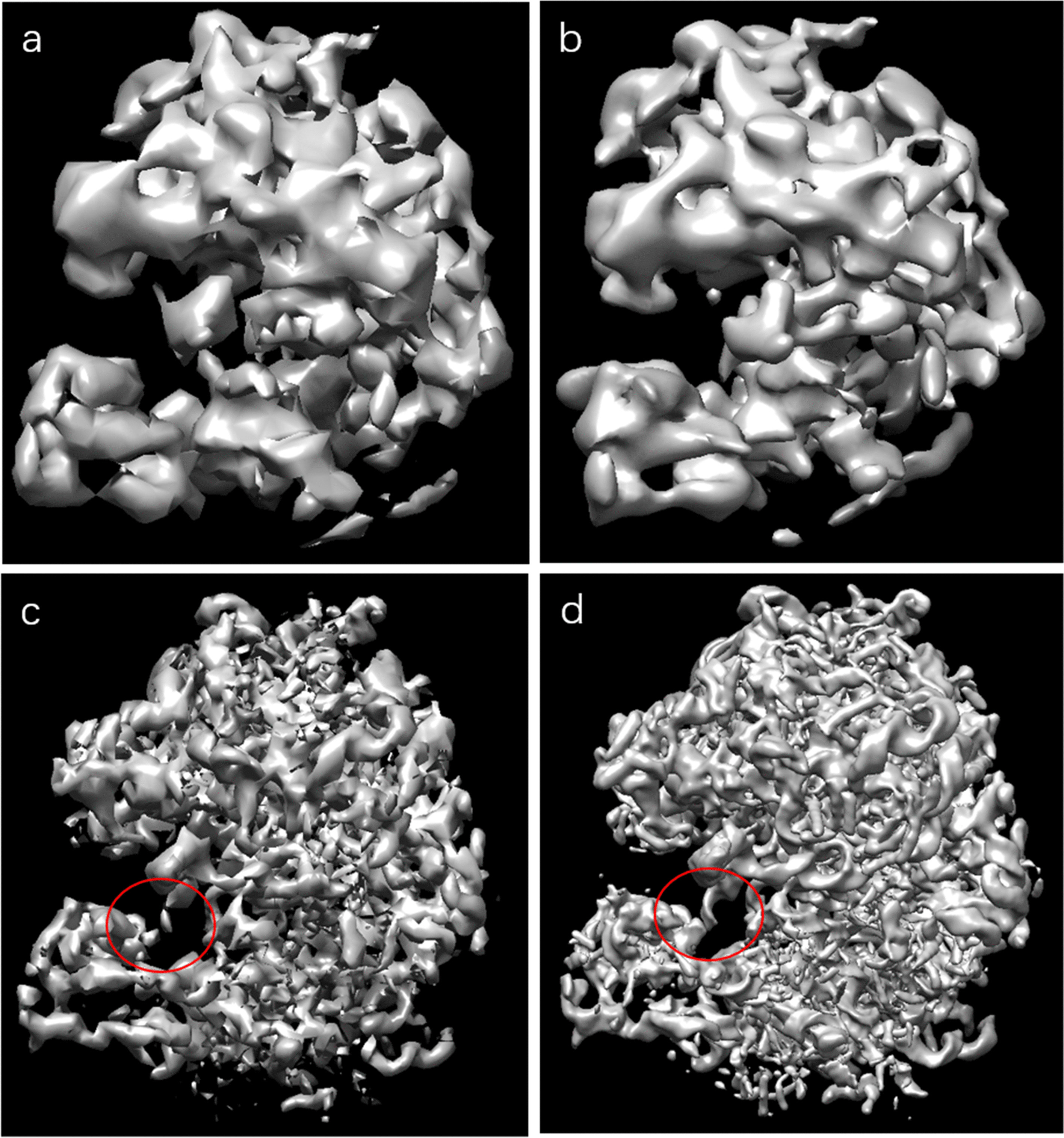
Comparison diagram of the 3D reconstructed model. **a** Reconstruct the initial structure result graph (8.75 angstrom) by Relion. **b** The initial model structure is restored by this method (7.46 angstrom). **c** The first round of 3D Refine effects (4.87 angstrom) is not preprocessed by the method of this paper. **d** The results of the first round of 3D Refine iterations (4.25 angstrom) of the method of this paper

In order to further verify the effectiveness of the proposed method in this paper, the class average is generated by using the classification method and alignment algorithm in this paper, and then the class average is used for initial three-dimensional reconstruction. The experiment used EMD5785 simulated cryo-single-particle cryo-EM images and real cryo-EM projection images in EMPIAR-10028. This experiment uses the ASPIAR software package (http://spr.math.princeton.edu/). The resulting class average is initially reconstructed into a three-dimensional structure using a covalent line reconstruction method based on the covalent line [35], which is implemented in the ASPIRE software package with the function $''cryo-estimate-mean''$. The projection matching algorithm is used to estimate the projection direction of the cryo-EM image, and the public line between the various types is estimated using the weighted voting algorithm we propose. All cryo-EM 3D structures are visualized by UCSF Chimera software. The results of each visualization are shown in Fig. 10 below. It can be seen from the comparison figure that the method proposed in this paper has effective reconstitution of 70 s ribosomes.

## Discussion

Our method tackles significant challenges that other 2D classification approaches have faced such as the difficulty of processing low SNR micrographs, the effectiveness of classification results to be improved, and the difficulty of calculating projection orientation information. In view of the problem of low SNR of micrographs, We incorporate noise reduction before clustering, and set a mask, which can effectively reduce the loss of delocalization information around particles, and retain more feature information while improving the SNR as much as possible. Aiming at the problem of low accuracy of classification results, we propose to use a deep clustering network based on contrast learning, which is mainly divided into two stages, the first stage is characterized learning through the comparative learning network, and the second stage integrates the characteristic information and weights of the first stage for clustering, and it is proved through experiments that the methods superior to the current deep learning can be obtained on both the simulated data set and the real data set. Aiming at the problem that projection orientation information is difficult to calculate, we propose to use a fast nearest neighbor interpolation method based on the maximum value of the frequency domain space, which can effectively estimate the rotation angle and translation alignment parameters in the x-axis and y-axis directions, which has an important role in evaluating the same type of data. However, the proposed method in this paper performs poorly on the classification of highly symmetrical structures, and it is difficult to estimate the rotation angle and orientation information.

## Conclusions

Our method performs best on ribosomes, which are easy to search for Fourier spatial angles due to the lack of high symmetry in the ribosome structure and the small molecular weight. Aiming at the problem that the training dataset cannot be labeled, the unlabeled cryo-EM single particles are classified by using the improved contrast learning clustering method, and pseudo-label data can be obtained for self-supervised training according to the probability vector of the second stage, which can be unaffected by molecular symmetry and obtain better classification results. Aiming at the problem that the projection direction of single particles of biological macromolecules is difficult to estimate, a fast maximum neighbor interpolation method based on frequency domain space is calculated by using the sample data in the class, which can effectively estimate the rotation angle and translation parameters. Finally, the aligned single-grain image is weighted to average according to SNR, thereby improving the result of class averaging. The above method can also be applied to real data sets, and experimental results show that SimCryoCluster performs as well as the most advanced method of single-particle 2D classification.

## Data Availability

The datasets used in this study and the source are availabel at https://www.ebi.ac.uk/empiar/.

## Abbreviations

Cryo-EM: Cryo-electron microscopy
Micrograph: Digital image taken through a microscope
MRC: Medical Research Council
PNG: Portable network graphic
